# Mullerian Adenosaroma of the Cervix with Sarcomatous Overgrowth and Heterologous Elements Presenting as a Recurrent Cervical Polyp

**DOI:** 10.1155/2012/358302

**Published:** 2012-08-16

**Authors:** Slim Charfi, Rim Kallel, Hela Mnif, Sameh Ellouze, Mohamed Dhouib, Mohamed Guermazi, Abdelmajid Khabir, Tahya Sellami-Boudawara

**Affiliations:** ^1^Laboratoire D'anatomie et de Cytologie Pathologiques, Department of Pathology, CHU Habib Bourguiba, 3029 Sfax, Tunisia; ^2^Department of Gynaecology and Obstetrics, CHU Hedi Chaker, 3029 Sfax, Tunisia

## Abstract

Mullerian adenosarcoma of the cervix is a rare tumor composed of benign epithelial and malignant stromal components. Sarcomatous overgrowth and heterologous elements in cervical adenosarcoma are extremely infrequent. We report the case of a 26-year-old woman admitted at the gynaecology department for a painless mass protruding from her vagina. The initial pathological exam concluded to endocervical polyp. Six months later, the patient was readmitted with a recurrence of the polyp. The pathological exam demonstrated interlacing fascicles of elongated spindle cells with few mitotic activity and no glandular formation. After reviewing of the initial polyp the diagnosis of mullerian adenosarcoma was suggested. A second recurrence of the polyp was noted one month later. Histopathological exam of the recurrent polyp confirmed the diagnosis of adenosarcoma with sarcomatous overgrowth and heterologous elements. The patient was lost for follow-up. Cervical adenosarcoma with sarcomatous overgrowth and heterologous element is a rare tumor that occurs in younger age in contrast to endometrium/corpus uterin mullerian adenosarcoma. In young women with recurrent cervical polyp, mullerian adenosarcoma must be considered and should be excluded by careful histopathological exam. Sarcomatous overgrowth and myometrial invasion are the most important prognostic factors. Treatment strategy is still unclear.

## 1. Introduction

Mullerian adenosarcoma (MA) is a rare variant of mullerian mixed tumor with low malignant potential. It is characterized by an intimate admixture of benign but sometimes atypical glandular epithelium and a sarcomatous stromal component, usually of endometrial stromal type with low grade features [1, 2]. MA occurs most often in the uterus and also in extrauterine sites, particularly the ovary. In uterus, most tumor involve the uterine corpus/endometrium, less frequently the uterine cervix. Sarcomatous overgrowth (SO) in MA is defined as the presence of pure sarcoma usually high grade and without a glandular component occupying at least 25% of the tumor [1, 2]. Heterologous elements are found in 22% to 24% of all MA [1, 3]. In cervix, MA with sarcomatous overgrowth and heterologous elements is an extremely rare entity [4–11].

## 2. Case Presentation

**Figure 1:**
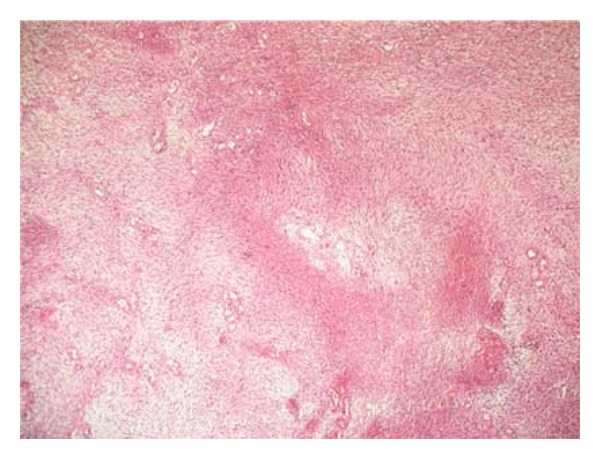
interlacing fascicles of elongated spindle cells with no glandular formations.

**Figure 2:**
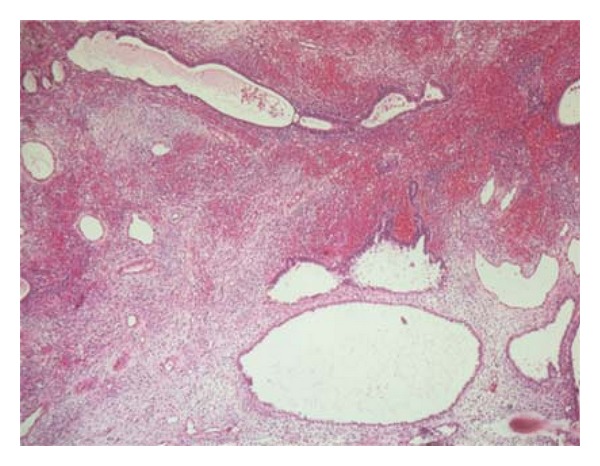
Endocervial glands surrounded by a cellular stroma with periglandular condensation.

**Figure 3:**
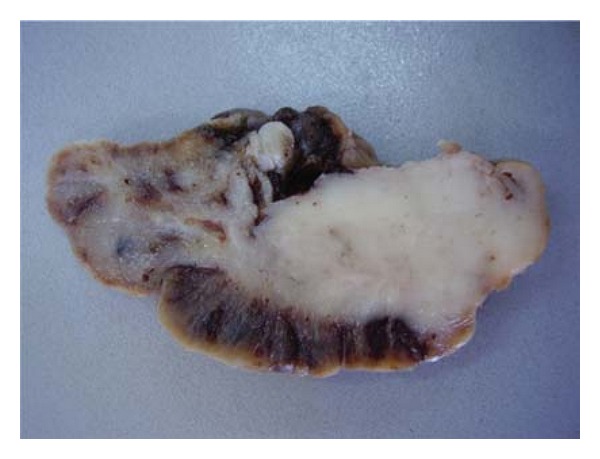
Irregular polypoid formation with white-grey to tan appearance. Note the presence of microcysts.

**Figure 4:**
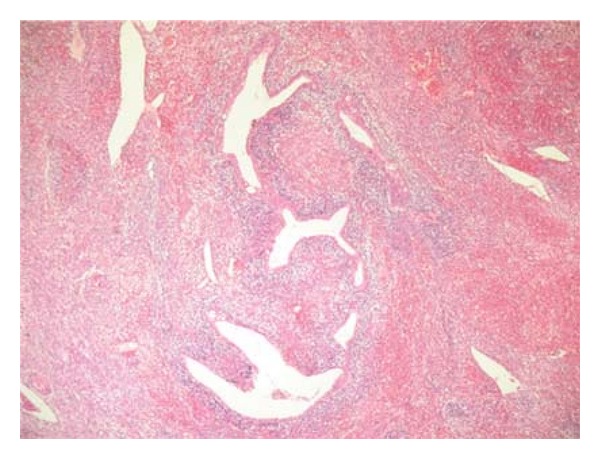
adenosarcoma with phyllodes-like appearance and dense periglandular stromal condensation.

**Figure 5:**
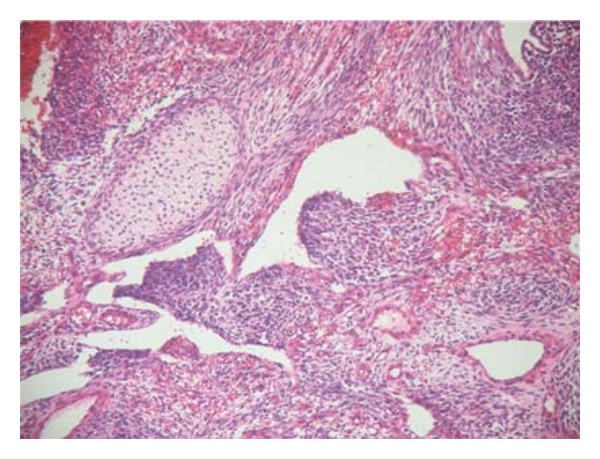
Cartilaginous foci in adenosarcoma.

**Figure 6:**
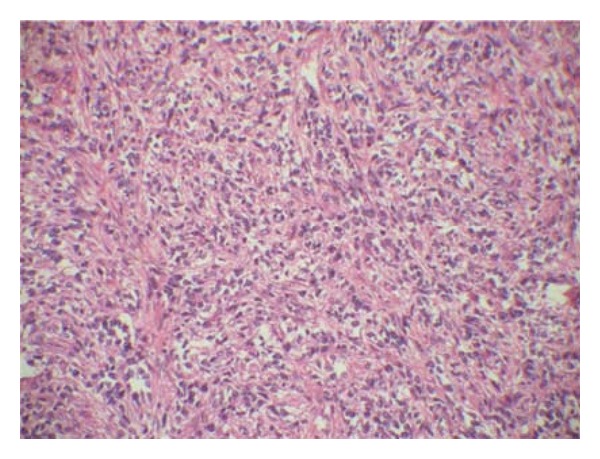
Adenosarcomatous overgrowth area in adenosarcoma: Stromal cells are large and pleomorphic.

A nulliparous and unmarried 26-year-old woman has been presented to the gynecological department with a painless mass protruding from her vagina. On examination, a 2 cm polyp was seen filling the vagina and exteriorised at the vulva. The polyp was removed and was diagnosed as endocervical polyp. Six months later, the patient was readmitted for a recurrence of the polyp witch was resected. On microscopic exam, the polyp was composed of interlacing fascicles of elongated spindle cells with only two mitotic figures per 10 high power fields (HPFs) (Figure 1). It was associated to ulcerations and inflammatory infiltrate. No glandular formation was found. Initial polyp was reviewed, and disclosed some focal increase in stromal cellularity with mild nuclear atypia (Figure 2). No mitotic activity was noted. The diagnosis of MA was suggested and a total hysterectomy was indicated. Before debuting the treatment, a second recurrence of the polyp was noted. The recurrent polyp measured 6 cm. At cut surface, it was solid with white-grey to tan appearance. Microcytic formations were observed (Figure 3). HThe histological exam showed an intimate admixture of benign appearing gland and sarcomatous stroma with areas of sarcomatous overgrowth (50% of the tumor). The glandular epithelium was primarily of endocervical type presenting phyllode-like features (Figure 4). Neither atypia nor mitotic activity was seen in this component. Sarcomatous areas around glandular component showed a low-grade endometrial stromal sarcoma appearance with small foci of benign cartilage (Figure 5). Nuclear atypia was low to moderate; mitotic rate was 4 per 10 HPFs. Areas of sarcomatous overgrowth displayed a range of appearance ranging from paucicellular with myxoid stroma to hypercellular epitheliod morphology (Figure 6). Mitotic activity was high (25 mitosis per 10 HPFs). There was no clear demarcation between low grade and high-grade stromal components. Immunohistochemical study demonstrates strong positivity of stromal cells for vimentin and desmin suggesting a rhabdomyosarcomatous differentiation. The patient was lost for follow-up. 

## 3. Discussion

MA is an infrequent neoplasm comprising benign glandular and a low-grade malignant stromal component in most cases [1, 2]. Cervical MA represents 2% to 9% of all MA locations. The younger age of presentation is more common in cervical AS than in uterine corpus. According to 55 cases of cervical MA, including our paper, mean age was 27 years [4–14]. The youngest patient was 10 years old [14]. In the review of Ramos et al., one-third of patients were under 15 years [4]. Sarcomatous overgrowth is defined as the presence of pure sarcoma, usually of high grade and without a glandular component, occupying at least 25% of the tumor [1]. MA with SO is reported in about 33% of uterine corpus MA and was associated in 67% of cases with recurrence [1]. In cervix, MA with SO are extremely rare; only four case reports were published [5, 6, 10, 11]. In uterine location, immunoreactions for two markers of cell proliferation, Ki-67 and p53 were stronger in MA with SO than in typical MA. In contrast the expression level of cell differentiation markers CD10 and Progesteron receptor was higher in typical AS than in AS with SO. Furthermore immunohistochemical findings in MA with SO are similar to findings in carcinosarcoma [1]. Heterologous elements in cervical MA are rarely reported [4–9]. They account for 8% to 42% of cervical MA [8, 9]. Most common elements are cartilage and striated muscle. Bone and lipoblast are less frequent. Cartilage elements, as in our case, are benign in most cases and presented as minor foci of cartilage [4, 7]. The presence of cartilage in cervix is also recognized in carcinosarcoma, embryonal rhabdomyosarcoma (ERMS), and mature teratoma. Recently Terada reported an undescribed endocervical polyp with cartilaginous metaplasia [15]. The differential diagnosis of cervical MA included embryonal (ERMS), carcinosarcoma, and adenofibroma. Botryoid ERMS resemble adenosarcoma because of polypoid growth characteristics and epitheliotropic stromal condensation (cambium layer). In contrast to ERMS, MA shows a phyllode-like architecture and intraglandular stromal projections. Immunohistochemical study cannot discriminative since both rhabdomyoblastic differentiation in MA and ERMS are positive for skeletal muscle markers. Carcinosarcoma can usually be distinguished from MA by the presence of high-grade malignant epithelial and mesenchymal components. Adenofibroma are rare and do not demonstrate stromal condensation around epithelium or high-mitotic activity and stromal atypia [2]. The distinction may be difficult. Recently, the study of Gallardo and Prat concluded that there is no evidence that there exists histopathological criteria that will reliably distinguish adenofibroma from AS. Thus these authors consider adenofibroma as well differentiated MA [1]. Cervical MA are in a significant proportion diagnosed, as our case, with a history of recurrent cervical polyp [3, 4, 9]. Kerner and Lichtig report seven cases of cervical AS misdiagnosed initially as benign cervical polyp [9]. These findings highlight the fact that recurrent polyp should be considered suspicious for adenosarcoma. Unfavorable prognostic factors for mullerian adenosarcoma are cytological atypia, high-mitotic rate, SO, presence of heterologous elements, dep myometrium invasion, necrosis and extra-uterine spread [8, 9]. Of these, SO and myometrial invasion are most relevant pronosis factors and are consistenly associated with poor outcome and recurrence [1]. Therapeutic options for MA are still undefined. Most authors recommend total hysterectomy. Local excision has been curative in rare cases [16]. Radiation with or without chemotherapy is reserved for tumors invading more than halfway through myometrium [13].

In conclusion, cervical MA with sarcomatous overgrowth and heterologous element is a rare tumor that occurs in younger age in contrast to endometrium/corpus uterin AS. In young women with recurrent cervical polyp, MA must be considered and should be excluded by careful histopathological exam. SO and myometrial invasion are the most relevant prognostic factors. Treatment strategy is still unclear.
